# Pseudoexfoliation Syndrome

**DOI:** 10.5005/jp-journals-10008-1148

**Published:** 2013-09-06

**Authors:** Murali Ariga, M Nivean, P Utkarsha

**Affiliations:** Senior Consultant, Department of Ophthalmology, Glaucoma Clinic, MN Eye Hospital, Chennai-600021, Tamil Nadu, India; Consultant, Department of Ophthalmology, MN Eye Hospital, Chennai, Tamil Nadu, India; Resident, Department of Ophthalmology, MN Eye Hospital, Chennai, Tamil Nadu, India

**Keywords:** Pseudoexfoliation, Weak zonules, Mydriasis, Secondary glaucoma, Homocysteine, Mutations.

## Abstract

Pseudoexfoliation (PXF) syndrome is a well-recognized clinical entity of considerable clinical significance. It is associated with poor mydriasis, cataracts with weak zonular support, secondary glaucoma and possibly with biochemical abnormalities, such as elevated homocysteine and systemic diseases involving the cardiovascular and central nervous system. There have also been some recent studies identifying mutations in genes which are associated with PXF.

**How to cite this article:** Ariga M, Nivean M, Utkarsha P. Pseudoexfoliation Syndrome. J Current Glau Prac 2013;7(3): 118-120.

## INTRODUCTION

**Fig. 1:**
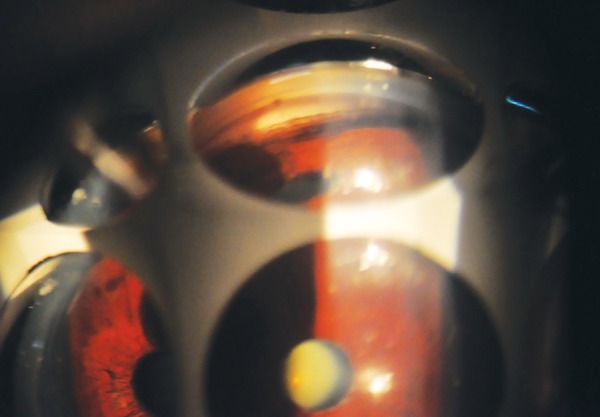
Goniophotograph showing increased TM pigmentation in PXF

**Fig. 2:**
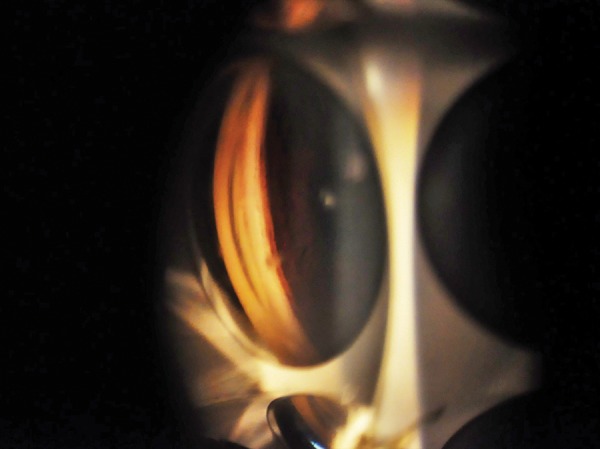
PXF material in angle

Pseudoexfoliation syndrome (PXF) was first described by a Finnish ophthalmologist in 1917. It is an age-related systemic disease having ocular manifestations (Fig. 1). It is characterized by deposition of a white fuffy amyloid-like proteinaceous material in the eye. The common sites of its deposition are the anterior chamber and its angle (Fig. 2), trabecular meshwork, anterior surface of iris, anterior capsule of lens and sometimes cornea.

The prevalence of PXF may vary in the population being studied and ranges from 6 to 10%. It may lead to secondary glaucoma which is relatively difficult to treat. Its incidence is reported to be more in women and increases with age. It is also reported to have familial predisposition. It is called ‘pseudoexfoliation' to differentiate it from true exfoliation which is due to heat or infrared related changes in anterior lens capsule.

PXF is a relatively common cause of chronic open angle glaucoma and this secondary glaucoma is known as pseudoexfoliation glaucoma. PXF is more common in females but males appear to be at greater risk of developing glaucoma if they have PXF.

A high-risk of developing PXF is conferred by mutations in the LOX1 gene at the locus 15q22, which codes for elastic fiber components of extracellular matrix. It is associated with some vascular disorders, hearing loss and Alzheimer's disease. Elevated plasma homocysteine, a risk factor for cardiovascular disease, is more common in exfoliation syndrome and exfoliative glaucoma patients than healthy controls.^1^

## CLINICAL FINDINGS

### Lens

**Fig. 3:**
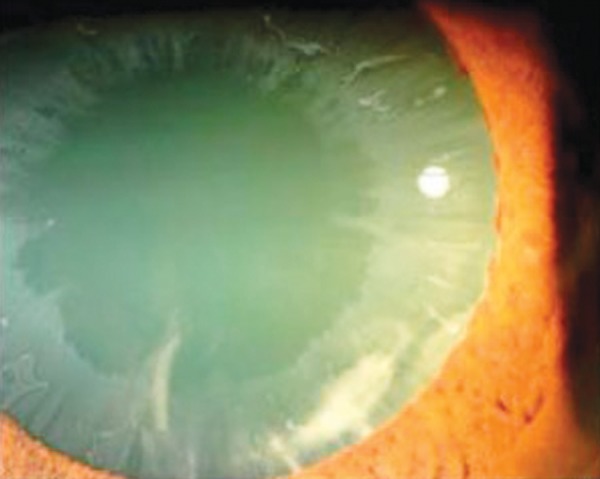
Classic pattern of PXF seen in the lens

Deposits of white material on the anterior lens surface are the most consistent and important diagnostic feature of PXF. The classic pattern consists of three zones: a central disk corresponding roughly to the diameter of the pupil; a granular, often layered, peripheral zone, and a clear area separating the two (Fig. 3).

The central zone is a homogeneous, white sheet and is often absent, while the peripheral zone is always present. The clear zone is created by rubbing of the iris over the surface of the lens during pupillary movement. Phacodonesis, or looseness of the lens because of damage to the zonules which hold the lens in place, is common and is one of the leading factors predisposing to an increase in complications at the time of cataract surgery. Spontaneous partial or complete dislocation of the lens can occur.

### Iris

**Fig. 4:**
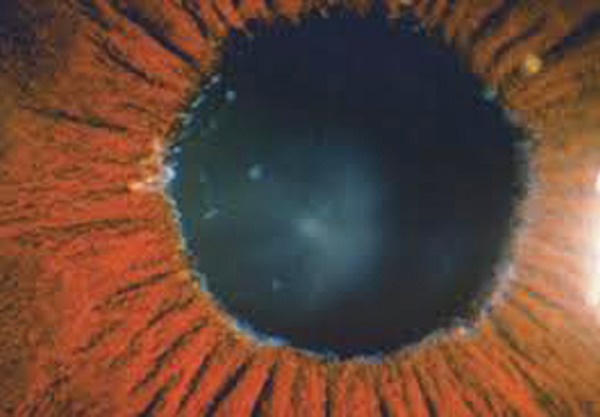
Fibrillar PXF material seen in pupillary border

Iris changes are an early and well recognized clinical feature in PXF. Next to the lens, exfoliation material is most prominent at the pupillary border (Fig. 4). Pigment loss from the iris sphincter region and its deposition on anterior chamber structures is a hallmark of PXF.

Loss of iris pigment and its deposition throughout the anterior segment are refected in iris sphincter region transillumination defects, loss of the pupillary ruff, pigment dispersion in the anterior chamber after pupillary dilation, pigment deposition on the iris surface, increased trabecular meshwork pigmentation and pigment deposition on the iris surface.^2^ The blood vessels of the iris are often narrowed and may become obliterated. In advanced stages, the cells of the vessel wall degenerate completely. Fluorescein angiographic studies have shown partial occlusion of radial iris capillaries associated with hypoperfusion, a reduced number of vessels, formation of tiny new blood vessels, and diffuse, patchy fuorescein dye leakage.

### Cornea

**Fig. 5:**
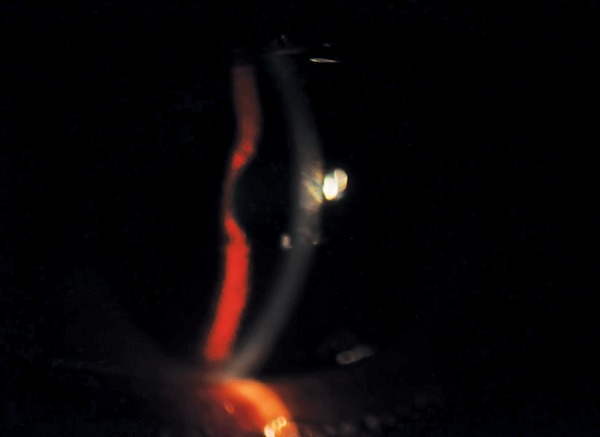
PXF material on corneal endothelium

Flakes of exfoliation material may be present on the endothelium (Fig. 5). There may be a diffuse, nonspecific pigmentation of the central endothelium, occasionally having the pattern of a Krukenberg spindle. Pigment is characteristically deposited on Schwalbe's line and sometimes as a wavy line or lines anterior to Schwalbe's line (Sampaolesi line). The number of corneal endothelial cells is reduced and central corneal thickness is also greater in eyes with PXF, perhaps refecting early corneal dysfunction.^3^ These changes predispose to early corneal decompensation at only moderate rises of intraocular pressure (IOP) or after cataract surgery.^4^

### Other Findings

Eyes with PXF often dilate poorly. Exfoliation material may be detected earliest on the ciliary processes and zonules, which are often frayed and broken. Abnormal zonular attachment to the lens or ciliary body may account for the development of lens subluxation or dislocation. Deposits of exfoliation material cover the crests of the ciliary processes in the pars plicata.

Increased trabecular pigmentation is a prominent sign of PXF and is apparent in virtually all patients with clinically evident disease (see Fig. 1). The distribution of the pigment tends to be uneven or splotchy and, in clinically unilateral cases, is almost always denser in the involved eye.

There appears to be a highly significant correlation between elevated IOP and the degree of pigmentation of the meshwork. In virtually all studies of patients with unilateral involvement, the trabecular pigment is almost always denser in the involved eye. Eyes with exfoliative glaucoma tend to have greater pigmentation than eyes with PXF but without glaucoma and eyes with exfoliative glaucoma have greater pigmentation than eyes with COAG. Marked IOP rises can occur in eyes with PXF after dilation for retinal examination. Postdilation IOPs should be checked routinely and the anterior chamber examined for pigment liberation in all patients receiving dilating drops.

### Ocular Associations

Increasing evidence suggests a causal association between PXF and cataract formation. The iris pigment epithelium and the lens surface, both coated with exfoliation material, tend to adhere (posterior synechiae), particularly when pupillary movement is inhibited by miotic therapy. Vigorous dilation can result in adhesion of the entire iris pigment epithelium onto the lens surface.

Patients with PXF are much more prone to have complications at the time of cataract extraction. Eyes with PXF dilate less well and have greater incidences of capsular rupture, zonular dehiscence and vitreous loss. Pupillary diameter and zonular fragility have been suggested as the most important risk factors for capsular rupture and vitreous loss.

Zonular fragility increases the risk of lens dislocation, zonular dialysis or vitreous loss up to 10 times. Postoperative complications of posterior capsular opacification, capsule contraction syndrome, intraocular lens decentration and infammation are also greater in eyes with PXF.

A possible association of PXF with retinal vein occlusion has also been suggested.^5^ In clinically unilateral cases of PXF, ipsilateral pulsatile ocular blood fow and carotid blood fow have been reported to be reduced. Eyes may be more prone to developing dry eye syndrome, especially if treated with b-adrenergic blocking agents.

### Pseudoexfoliative Glaucoma

Pseudoexfoliative material blocks spaces in the trabecular meshwork, promoting the accumulation of pigment and cellular debris. This causes obstruction of channels through which aqueous humor normally outfows into Schlemm's canal*.* This is believed to be the causative factor for chronic elevations of IOP and the development of pseudoexfoliation glaucoma.

In unilateral exfoliation syndrome, IOP of the exfoliative eye is approximately 2 mm Hg higher than IOP of the nonexfoliative fellow eye. Glaucoma in the exfoliation syndrome has been shown to have a more serious clinical course than in primary open-angle glaucoma (POAG). At the time of diagnosis, IOP and its diurnal variation are generally higher and visual field defects tend to be greater in exfoliation glaucoma than in POAG.^6^ Also, normotensive fellow eyes of patients with unilateral high-tension PXF glaucoma are under significant risk of glaucomatous damage, related with the level and the fuctuation range of IOP.^7^

### Extraocular Findings

Aggregates of exfoliation fibers have been identifed in skin and in autopsy specimens of heart, lung, liver, kidney, gall bladder and cerebral meninges in two patients with ocular PXF.^8^ The deposits were focally present in the interstitial fibrovascular connective tissue septa of these organs, frequently adjacent to elastic fibers, elastic microfibrils, collagen fibers, fibroblasts and to the walls of small blood vessels.

In the Blue Mountains Eye Study (Australia), PXF correlated positively with a history of hypertension, angina, myocardial infarction or stroke, suggestive of vascular effects of the disease.^9^ In another study, 50% of persons with PXF had cardiovascular disease, three times the rate of the unaffected subjects. In yet another study, PXF was significantly associated with aneurysms of the abdominal aorta, but not with carotid artery occlusion.
